# The Immune Change of the Lung and Bowel in an Ulcerative Colitis Rat Model and the Protective Effect of Sodium Houttuyfonate Combined With Matrine

**DOI:** 10.3389/fimmu.2022.888918

**Published:** 2022-06-30

**Authors:** Lulu Ni, Shan Jing, Li Zhu, Xue Yang, Xinyue Wang, Su Tu

**Affiliations:** ^1^ Department of Basic Medicine, Wuxi School of Medicine, Jiangnan University, Wuxi, China; ^2^ Department of Internal Medicine of Traditional Chinese Medicine (TCM), Nantong Hospital, Nantong, China; ^3^ Department of Internal Medicine of Traditional Chinese Medicine (TCM), Dong- zhimen Hospital, Beijing University of Chinese Medicine, Beijing, China; ^4^ Department of Internal Medicine of Traditional Chinese Medicine (TCM), Third Affiliated Hospital of Henan University of Traditional Chinese Medicine, Zhengzhou, China; ^5^ Department of Emergency, the Affiliated Wuxi NO 2 People’s Hospital of Nanjing Medical University, Wuxi, China

**Keywords:** ulcerative colitis, lung injury, inherent immunity, adaptive immunity, sodium houttuyfonate combined with matrine

## Abstract

**Objective:**

To explore the immune change of lung injury of Ulcerative colitis (UC) by observing the changes of inherent immunity and adaptive immunity of the lung and bowel in UC rat models after the treatment of Sodium Houttuyfonate combined with Matrine.

**Method:**

UC rat models were established with the mucous membrane of colon allergize combined with TNBS-alcohol enteroclysis for 1 week and 5 weeks. 1-week experimental rats were divided into normal group and model group, 5/each group. 5-weeks experimental rats were divided into normal group, model group, Sodium Houttuyfonate (2.9mg/ml) combined with Matrine (1.47mg/ml), and positive control sulfasalazine (10mg/ml), 5/each group. All rats were administered by gavage for 5 weeks. The histopathological and fibrotic changes in the lung and bowel were observed, and the expressions of Tumor Necrosis Factor (TNF)- α, interleukin (IL)-8 in the lung, bowel, and serum were detected by radio-immunity and immunohistochemistry, and the mRNA expressions of Toll-like receptor (TLR)-4, nuclear factor kappa (NF-κB), Macrophage migration inhibitory factor (MIF), Mucosal addressing cell adhesion molecule-1 (MadCAM1) and Pulmonary surfactant protein-A (SP-A) in the lung and bowel were detected by Real time-PCR.

**Result:**

Compared with the normal group, the model rats had significant histopathological and fibrotic changes both in the lung and bowel, and all treatment groups were improved. After treatment, TLR4, IL-8, MIF, and TNF-α in the lung decreased (P<0.05); NF-KB, IL-8, and MIF in the bowel increased (P<0.05); MadCAM1 both in lung and bowel decreased (P<0.05); SP-A decreased in bowel and increased in the lung (P<0.05).

**Conclusion:**

The cause of lung injury in this model was found to be related to inherent immunity and adaptive immunity, while the cause of bowel injury in this model was found to be mainly related to adaptive immunity. Sodium Houttuyfonate combined with Matrine could improve bowel and lung injury.

## Introduction

Ulcerative colitis (UC) and Crohn’s disease (CD) are the two main kinds of inflammatory bowel disease (IBD). They might be related to heredity, environment, diet, psychology, and immune disorders but the etiology remains unknown. UC is characterized by chronic, non-specific inflammation and ulceration of colon mucosa. Extraintestinal manifestations of UC are very common, these inlcude dermatological manifestations, erythema nodosum and pyoderma gangrenosum, ocular manifestations, uveitis and episcleritis, hepatobiliary manifestations, primary sclerosing cholangitis and autoimmune hepatitis, musculoskeletal manifestations, peripheral arthritis, and axial arthropathy (1). In contrast, pulmonary manifestations are rare, including bronchiectasis (2), pulmonary vasculitis (3, 4), bronchiolitis obliterans-organizing pneumonia (BOOP) (4, 5), and other lung diseases. Pulmonary abnormality might be correlated with autoimmune disorders (6, 7). This intriguing shift of the inflammatory process from the bowel to the lung is often taken as evidence of a causal link between the two processes, probably because of the common embryological origin of the intestinal and pulmonary mucosa (8). The relationship between the respiratory system and the colon is complex. In modern medicine, researchers have found a close relationship between the lung and the bowels through studies investigating embryonic development (9), mucosal biofilms (10), and the mucosal immune system (11). The mucosal immune system is comprised of both the gastrointestinal and respiratory mucosae. Mucosal lesions occurring in these organs likely affect others through the mucosal immune pathway. In histology and embryology, both colonic and respiratory epithelial cells originated from the primitive foregut. Yilmaz hypothesized the pathophysiology of the respiratory damage in IBD wherein the presence of goblet cells and submucous glands were observed in both the colonic and respiratory epithelial cells (12). Furthermore, in immunology, the respiratory and gastrointestinal tracts shared common submucosal lymphoid tissues that played an important role in the mucosal defense system of the host. Therefore, the pathological similarities shared between the two structures result in increased similarities of the mucosal immune system (13). In our previous study, we found a pathological connection between the lung and bowel. The longer the course of disease, the greater the scope of the disease, and the more serious the pulmonary function damage in patients with UC (14). Our previous animal experiments have also found that UC rat models showed the presence of lung pathological injury.

For two millennia, Traditional Chinese Medicine (TCM) has been used to treat various diseases. Currently, some studies have reported its effectiveness in the management of UC (15). *Houttuynia cordata* have been used to prevent and treat lung-related diseases and alleviate symptoms by TCM practitioners, including cough and dyspnea, for centuries. For this study, we selected Sodium Houttuyfonate, a particularly stable compound of *Houttuynia cordata* extract. Studies showed that Sodium Houttuyfonate had significant antibacterial and anti-inflammatory effects (16). Additionally, Matrine, extracted from the roots of So*phora flavescens* has demonstrated its effectiveness in the treatment of UC and lung injury (17)due to its biological properties including anti-inflammatory, antioxidative, and antimicrobial activities (18). In another study, Oxymatrine (opioid agonist) ameliorated the congestion and erosion in luminal mucosa and improved the IBD symptoms induced by inflammation (19). Considering these, UC-related lung injury could potentially be improved by Sodium Houttuyfonate combined with Matrine.

The immune response and inflammatory pathway of UC showed that the cytokine network is an important part of UC formation (20). Wang reported on the blocked TLR4/NF- κB/MAPK pathway and promoted an anti-inflammatory response mediated by IL-10/JAK1/STAT3, which had protective effects on UC (21). NF-κB played a very important role in UC (22, 23). In lung injury, inhibited TLR4/NF-κB, reduced inflammatory mediators, and could prevent LPS induced acute lung injury (24, 25). IL-8 was a good predictor of lung injury in both adults (26) and fetuses (27), and its role in UC was also very significant (28–30). TNF - α plays an important role in the pathogenesis of UC (31, 32), and its role in the lung is becoming more prominent (33). In this research, we established UC rat models with immune-TNBS-ethanol and used Sodium Houttuyfonate combined with Matrine to treat UC-related lung injury. We detected the immune index, such as the above-related cytokine, chemotactic factor, adhesion molecule, and signal transduction molecule in the lung and bowel, to investigate the intervention mechanism and protective effect of Sodium Houttuyfonate combined with Matrine on innate immunity and adaptive immunity in a UC model for lung disease.

## Materials and Methods

### Animals

This study used 2-month-old male Wistar rats weighing 200 ± 10 g purchased from the Academy of Military Medical Laboratory Animal Centre (Beijing, China). Ten male New Zealand rabbits, weighing about 3 kg, were purchased from Haidian Thriving Animal Farm (Beijing, China). All animals were housed at SPF Animal Laboratory of Dongzhimen Hospital (Beijing, China) with free access to food and water and were kept in a regulated environment (23 ± 2°C) under a 12-hour light/dark cycle (with light turning on at 8:00 am). All animal procedures were reviewed and approved by the Animal Experimentation Committee at the Beijing University of Chinese Medicine before and during the experiment (Approval No. 18-08-2).

### Induction of Ulcerative Colitis

#### Antigen Preparation

Ten rabbits were killed by air embolism, then the colon was removed immediately and rinsed with sterile saline, and scraped the mucosa. The mucosa was mixed with equal saline and homogenized 30 times with a tight Dounce homogenizer (Sigma, USA). Samples were further disrupted by intermittent sonication (six 30 s pulses with a 1 min cooling period in between) and then centrifuged at 3,000 rpm (Eppendorf, Germany) for 30mins at 4°C. The supernatant was then aliquoted and stored at -20°C. The protein concentration was measured by bicin choninic acid (BCA) assay (CoWin Bioscience, China).

#### Antigen Preimmunization and Immune-2, 4, 6-Trinitrobenzene Sulfonic Acid Enema

Rabbit intestinal mucosal antigen solution was mixed with an equal volume of Freund’s complete adjuvant, which was used to prepare the antigen emulsion. Wistar rats were immunized with the antigen emulsion in the paws and groin alternately on day 1, day 15, and day 22. Each immune volume contained 8 mg of antigen protein per rat. On the 28th night, the rats fasted and were water-deprived overnight. The next day, the rats were anesthetized with 1% pentobarbital sodium. A medical-grade polyurethane cannula for enteroclysis was inserted into the anus, and the tip was advanced to 8 cm proximal to the anal verge. Immune-2, 4, 6-trinitrobenzene sulfonic acid (TNBS) (100 mg/kg) (Sigma, San Francisco, CA, USA) dissolved in 50% ethanol was rapidly instilled into the colon. Antigen preparation, antigen preimmunization, and TNBS enema were done as previously described (Liu et al., 2013). UC models were established with the mucous membrane of colon allergize, combined with TNBS-alcohol enteroclysis for 1 week and 5 weeks. 1-week experimental rats were divided into the normal group and model group, 5/each group. 5-week experimental rats were divided into normal group, model group, Sodium Houttuyfonate (2.9mg/ml) combined with Matrine (1.47mg/ml) and positive control sulfasalazine (10mg/ml), 5/each group. Oral needle intragastric administration was for 5 weeks. All experimental rats receiving intragastric administration for 5 weeks were euthanized by CO2 asphyxiation.

### Histopathological and Fibrotic Changes in the Lungs and Bowel

Colon tissues 2-10cm above the anus, lung, liver, and kidney tissue of the rats were taken and soaked in 4% paraformaldehyde solution. The 4% paraformaldehyde solution was changed every day. One week later, the fixed specimens were routinely dehydrated with alcohol gradient, transparent xylene, embedded with paraffin, and sectioned at 5 m thick. Two sections were taken from each rat for HE staining and Masson staining. Then the morphological changes in lung and intestinal tissues were observed and photographed under the light microscope.

### Radioimmunoassay and ELISA

All rats fasted for one night before euthanasia. After anesthetizing with 1% pentobarbital sodium administered peritoneally, blood was drawn from the abdominal aorta and the blood serum was stored in liquid nitrogen. All rats were subsequently euthanized using CO2 asphyxiation. Additionally, lung tissue was harvested and washed with normal saline, which was absorbed onto a filter paper, and was stored in liquid nitrogen. During the procedure, five times the amount of water was added to the lung. The lung tissue was disrupted using an ultrasonic disintegrator by intermittent sonication (six 30 s pulses with a 1 min cooling period interval). The sample was centrifuged at 3,000 rpm for 20 min at 4°C. The supernatant was collected, and TNF-α and IL-8 were detected in the lung tissue of rats in each group with a Radioimmunity kit (Beijing Kangyua Ruide Biotechnical Co. Ltd., Beijing, China), the MadCAM1 in the serum was detected with ELISA kit (Beijing Kangyua Ruide biotechnical Co. Ltd.).

### Real-Time Polymerase Chain Reaction Analysis

Total RNA from lungs and bowel were extracted using the TRZol reagent extraction kit (Invitrogen, CA, USA). The mRNA expressions of TLR4, NF-κB, MIF, MadCAM1, and SP-A in the lung and bowel were detected by Realtime-PCR. The sequences of the PCR primers were shown in **Table 1**. Ct of every sample was analysed by MxPro-Mx3000p software. The relative amount of each gene was calculated by utilizing the expression of GAPDH as internal control and using the equation 2^△△Ct^ where ΔCt = (Ct gene-Ct GAPDH). △△Ct=△Ct−average (△Ct normal), cDNA=2^△△Ct^. All Real-time PCR experiments were performed with Pwer SYBR Green PCR Master Mix (Applied Biosystems company, USA).

**Table 1:** PCR Primer(s).

Gene	Sequence of primers (5’ -3’)	Product Size	Cycling parameters
TLR4	F:CTT TCA GGG AAT TAG GCT CC R: CCA AGA TCA ACC GAT GGA C	116 bp	10min at 95°C, 30sec at 95°C, 30sec at 55°C, 20 sec at 72°C, for 40 cycles
NF-κB	F:ATC TGT TTC CCC TCA TCT T R: GTG CGT CTT AGT GGT ATC TG	167 bp	10min at 95°C, 30sec at 95°C, 30sec at 55°C, 20 sec at 72°C, for 40 cycles
MIF	F:TCT CCG CCA TGC CTA TG R:GGG TCG CTC GTG CCA CTA AA	178 bp	10min at 95°C, 30sec at 95°C, 30sec at 60°C, 20 sec at 72°C, for 40 cycles
MadCAM-1	F:CCG AAA TCC ACC AGA ACC R: TCC AAT GCA CCG TCA CTC	81 bp	10min at 95°C, 30sec at 95°C, 30sec at 54°C, 20 sec at 72°C, for 40 cycles
SP-A	F:CCA GAG CAG GAG GCA ACA T R:AGA AGC CCC ATC CAG GTA G	149 bp	10min at 95°C, 30sec at 95°C, 30sec at 58°C, 20 sec at 72°C, for 40 cycles
GAPDH	F: CCA TGG AGA AGG CTG GG R: CAA AGT TGT CAT GGA TGA CC	195 bp	10min at 95°C, 30sec at 95°C, 30sec at 54°C, 20 sec at 72°C, for 40 cycles

### Immunohistochemistry Analysis

Immunohistochemistry was used to demonstrate the expression and localization of IL-8 and TNF-α. The lung and bowel sections of rats in each group were incubated with IL-8 (rabbit anti-rat, 0.1 mg/mL, Abcam, Cambridge, UK) or TNF-α (rabbit anti-rat, Abcam, Cambridge, UK). They were detected using an undiluted horseradish peroxidase-conjugated polymer goat anti-rabbit immunoglobulin G polyclonal polymer as the secondary antibodies (CoWin Bioscience, Beijing China).

### Western Blot Analysis

The lung and bowel were then lysed with 200μl RIPA lysis buffer, protein contents were quantitated using a BCA protein reagent assay kit (CoWin Bioscience, China) and analyzed by 10-12% of SDS-PAGE, followed by immunoblotting using enhanced chemiluminescence substrate (Merck Millipore) according to the manufacturer’s instructions. The protein expressions of SP-A in the lung and bowel were detected by Western Blotting. Bands were visualized using a chemiluminescent detection system (ProteinSimple, San Jose, CA, USA).

### Statistical Analysis

Quantitative data were expressed as means ± standard deviations. The significant difference between groups was assessed using the Student’s t-test and one-way analysis of variance. *Post hoc* comparisons were made using the nonparametric Dunn multiple comparison test. In all tests, statistical significance was set at P < 0.05. Statistical analyses were performed using SPSS Software 13.0 (SPSS Incl, Chicago, IL, USA).

## Results

### Pathological and Fibrosis Changes of Lung and Intestinal Tissues in Rats With Ulcerative Colitis

**Figure 1:**
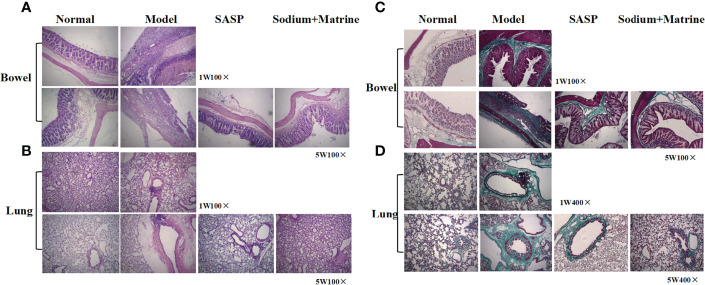
Pathological and fibrosis changes of lung and bowel tissues in rats with ulcerative colitis. UC models were established with the mucous membrane of colon allergize combined with TNBS-alcohol enteroclysis. **(A)** Intestinal and **(B)** lung tissues with HE staining; **(C)** Intestinal and **(D)** lung tissues with Ma Song dyeing.

At 1 and 5 weeks after modeling, the intestinal mucosa, muscularis mucosa, submucosa, and muscularis tissues of UC model rats had extensive inflammatory infiltration dominated by lymphocytes. The colonic wall showed coagulative transmural necrosis, and part of the necrotic tissue fell off and formed ulcers, with necrotic tissue covering the surface of the ulcer and new granulation tissue hyperplasia at the bottom. The peripheral mucosal structure was disordered, mucosal lamina propria gland atrophied or even disappeared, and there was submucosal and muscular vascular dilation (**Figure 1A**). Compared with the normal lung, extensive lymphocyte infiltration was observed in the lung tissues of the model group, and lymphocyte nodules were formed around the bronchi. Pulmonary septal thickening, alveolar space narrowing, alveolar fracture fusion, vascular wall, and bronchial wall also had obvious hyperplasia (**Figure 1B**). Sodium Houttuyfonate combined with the Matrine group improved with differing degrees of pathological changes and fibrosis (**Figures 1A, B**).

The intestinal mucosa of the normal group was normal with no inflammatory cell infiltration and only a small amount of collagen tissue. After 1 week of modeling, there was a small amount of collagen hyperplasia on the necrotic surface and basement of intestinal mucosa in the model group, and 5 weeks later, collagen hyperplasia in the basement of intestinal mucosa in the model group was significantly increased (**Figure 1C**). The bronchi of the normal group were normal, with no inflammatory cell infiltration and only a small amount of collagen tissue around the bronchi. The model group had a large number of inflammatory cells infiltration around the bronchi, and collagen tissue hyperplasia was significantly increased compared with the normal group, accompanied by erythrocyte exudation and bronchial smooth muscle hyperplasia (**Figure 1D**). The collagen tissue hyperproliferation in SASP and Sodium Houttuyfonate combined with Matrine treatment groups were increased compared to the normal group but significantly decreased relative to the model group (**Figures 1C, D**).

### The Changes in Inherent Immunity in the Lung and the Bowel

**Figure 2:**
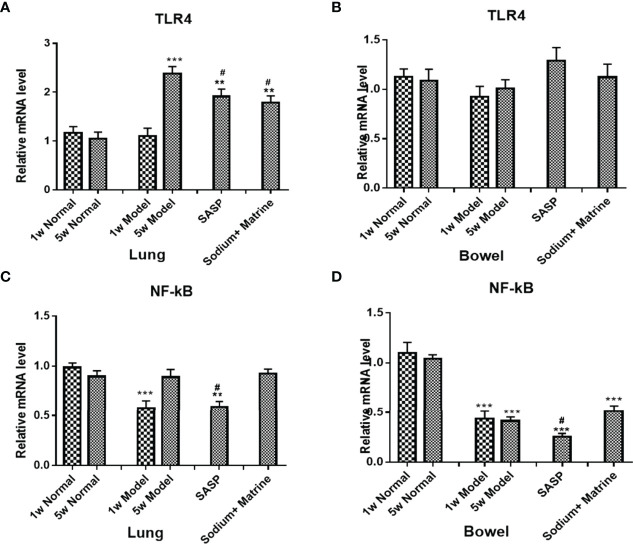
Expression of TLR4 mRNA, NF-kB mRNA, and MIF mRNA in the lung and bowel. UC models were established with the mucous membrane of colon allergize combined with TNBS-alcohol enteroclysis. TLR4 mRNA in Lung **(A)** and Bowel **(B)**; NF-kB mRNA in Lung **(C)** and Bowel **(D)**. Compared with Normal group, **P < 0.01; ***P < 0.001. Compared with model group, ^#^P < 0.05.

To examine the changes in the inherent immunity system in the lung and bowel, TLR-4 mRNA, NF-κB mRNA, TNF-α protein, IL-8 protein, and MIF mRNA were detected. TLRs are pattern recognition receptors of enter-mucosa inherent immunity recognizing pathogen-associated molecular patterns (PAMPs). Ligand binding to TLRs initiates signaling cascades that activate NF-κB, MAPK, and interferon response factors. The result showed that the expression of TLR4 mRNA in the lung of the model group had no significant deviation at 1 w (P>0.05), but increased significantly at 5 w (P<0.001); TLR4 mRNA significant decreased in both SASP and Sodium Houttuyfonate combined with Matrine groups (P<0.05) (**Figure 2A**). In the bowel of the model group, the expression of TLR4 mRNA dropped both at 1 w and 5 w, but had no significant difference (P>0.05); TLR4 mRNA in treatment groups slightly increased, but also had no significant difference (P>0.05) (**Figure 2B**). The expression of NF-κB mRNA in the lungs of the model group decreased significantly at 1 w (p<0.01), but had no significant differences at 5 w. There was no significant difference between the groups (**Figure 2C**) but in the bowel of the model group, the expression of NF-κB mRNA dropped significantly at 1 w and 5 w (p<0.01) (**Figure 2D**). The SASP group was down-regulated, while Sodium Houttuyfonate combined with the Matrine group was upregulated, but there was no significant difference (P>0.05).

**Figure 3:**
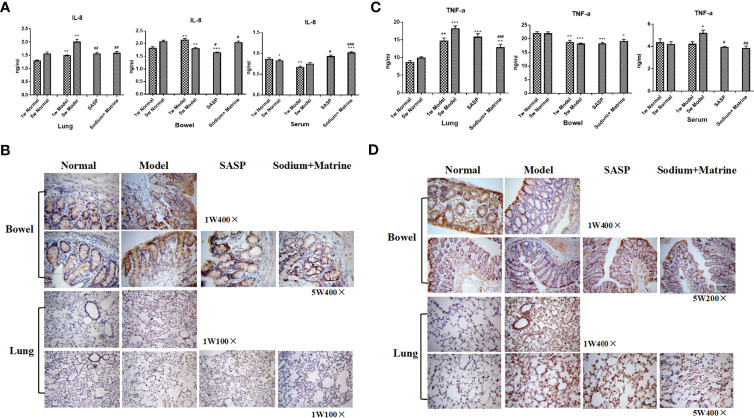
Expression of TNF-a, IL-8 in the lung, bowel, and serum. UC models were established with the mucous membrane of colon allergize combined with TNBS-alcohol enteroclysis. **(A)** Expression of TNF-a (Radioimmunoassay); **(B)** Expression of IL-8 (Radioimmunoassay); **(C)** Expression of TNF-a (Immunohistochemistry); **(D)** Expression of IL-8 (Immunohistochemistry). Compared with Normal group, *P < 0.05; **P < 0.01; ***P < 0.001. Compared with model group, #P < 0.05, ##P < 0.01 , ^###^P < 0.001.

TNF-α is mainly produced by lipopolysaccharide (LPS) activated mononuclear macrophages, and in inflammation, promotes neutrophil aggregation and activation, promotes endothelial cell adhesion molecule expression and promotes the inflammatory to start and continue. IL-8 is mainly produced by mononuclear macrophages, endothelial cells, skin cells, and T cells under the stimulus of IL-1, TNF-α, and LPS in an inflammatory reaction, and then causes tissue damage mainly through chemotaxis and activation of neutrophils, and chemotaxis of some basophils and T cells. The result showed that IL-8 in the lung of the model group increased significantly both at 1 w and 5 w (P<0.01), and in the bowel of the model group, IL-8 increased significantly at 1 w (P<0.01), but decreased at 5 w (P<0.05). In the serum of the model group, IL-8 significantly decreased at 1 w and 5 w (P<0.05). IL-8 increased in bowel and serum and decreased in the lung after Sodium Houttuyfonate was combined with Matrine treatment (**Figures 3A, B**). TNF-α in the lung of the model group increased rapidly both at 1 w and 5 w (P<0.01), but decreased significantly in the bowel of the model group both at 1 w and 5 w (P<0.05, P<0.01). TNF-α in the serum had no significant differences at 1 w (P>0.05) but increased at 5 w (P<0.05). TNF-α was significantly down-regulated in lung and serum after Sodium Houttuyfonate combined with Matrine treatment (P<0.01) (**Figures 3C, D**).

**Figure 4:**
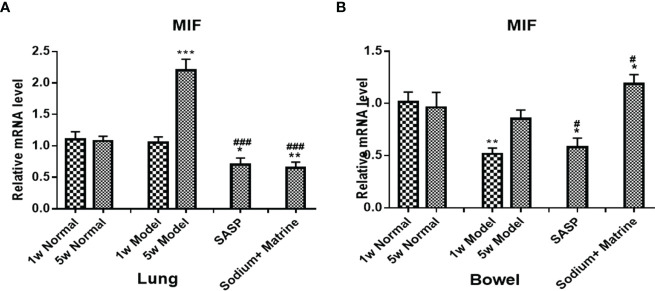
Expression of MIF mRNA in the lung and bowel. UC models were established with the mucous membrane of colon allergize combine with TNBS-alcohol enteroclysis. MIF mRNA in the lung **(A)** and bowel **(B)**. Compared with Normal group, *P < 0.05; **P < 0.01; ***P < 0.001. Compared with model group, ^#^P < 0.05, ^###^P < 0.001

MacroPhage migration inhibitory factor (MIF) is also an important proinflammatory factor, secreted by a series of immune cells, such as macrophages, dendritic cells, lymphocytes, neutrophils, and pituitary cells (34), and can be induced by LPS, interferon gamma (IFN-gamma), and TNF-α. The expression of MIF mRNA in the lungs of the model group have no significant deviation at 1 w (P>0.05) but increased significantly at 5 w (p<0.001). All treatment groups could significantly downregulate the expression of MIF (p<0.001) (**Figure 4A**). The expression of MIF mRNA in the bowel of the model group decreased significantly at 1 w (p<0.01), but had no significant deviation at 5 w (P>0.05). All groups were significantly upregulated compared with the model group (p<0.05), especially in Sodium Houttuyfonate combined with the Matrine group (**Figure 4B**). This suggests that the acute inflammation of the lung is mainly caused by TNF-α and IL-8, which was chemotaxis and activation of neutrophils. The decrease of NF-κB mRNA at the early stage of pneumonia could be caused by cell apoptosis. However, with the decline of cell apoptosis, TLR4 mRNA in the lung increased at 5 weeks, activating more NF-κB, which had no significant difference compared with the control. MIF mRNA increased remarkably at 5 weeks, which suggested that macrophages participate in the lung inflamation. The decrease of NF-κB and TNF-α could be caused by cell apoptosis, as necrotic tissue could be seen under the microscope, meaning they were not damage factors in the colitis. MIF decreased but IL-8 increased at 1 week, suggesting neutrophils but not macrophages work effectively in acute colon inflammation. Both MIF had no significant differences and IL-8 decreased in the bowel at 5 w (P<0.05), suggesting that neither neutrophils nor macrophages were involved in chronic colon inflammation.

### The Changes in Adaptive Immunity in the Lung and the Bowel

**Figure 5:**
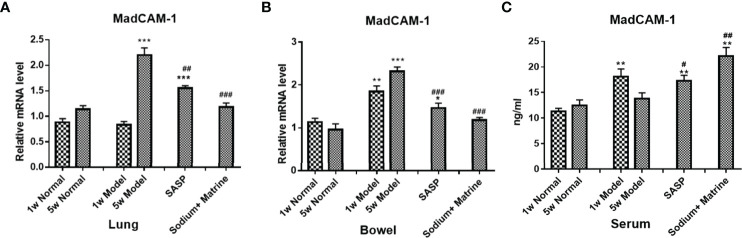
The Expression of MadCAM1 mRNA in the lung and bowel, and the Expression of MadCAM1 protein in the serum. UC models were established with the mucous membrane of colon allergize combined with TNBS-alcohol enteroclysis. MadCam-1 mRNA expression in lung **(A)** and bowel **(B)** (PCR); **(C)** MadCam-1 protein expression in surem (Elisa). Compared with Normal group, *P < 0.05; **P < 0.01; ***P < 0.001. Compared with model group, ^#^P < 0.05, ^##^P < 0.01, ^###^P < 0.001.

Adaptive immunity mainly contains CD8 + T cells and T killing cells (TK1). The mucosal addressin cell adhesion molecule 1 (MadCAM-1) interacts with its receptor (Alpha 4 beta 7 integrin) α4β7 integrin, which is highly expressed on CD8 + T cells and TK1, which are involved in lymphocytes homing to mucosal sites and cell-cell interactions during the immune response (16). In this model, the expression of MadCAM1 mRNA in the lung of the model group had no significant deviation at 1 w (P>0.05) but increased significantly at 5 w (p<0.001). All treatment groups could significantly reduce MadCAM1 expression (**Figure 5A**). The expression of MadCAM1 mRNA in the bowel of the model group increased significantly at both 1 w and 5 w (p<0.01), all treatment groups could significantly reduce MadCAM1 expression (**Figure 5B**). The expression of MadCAM1 protein in the serum increased significantly at 1 w (p<0.01), but had no significant deviation at 5 w (P>0.05), Sodium Houttuyfonate combined with Matrine significantly increased the content of MadCAM1 in serum (**Figure 5C**). MAdCAM-1 mRNA increased remarkably in the lung at 5 weeks, which suggested that lymphocytes were selectively homing to the lung during the period of chronic inflammation, and Sodium Houttuyfonate combined with Matrine could effectively improve the pulmonary inflammatory response. MAdCAM-1 increased both at acute and chronic colitis, suggesting lymphocytes of the adaptive immune system could account for the main damage factors of the bowel in this model, while Sodium Houttuyfonate combined with Matrine could alleviate this inflammatory damage.

### The Changes of SP-A in the Lung and the Bowel

**Figure 6:**
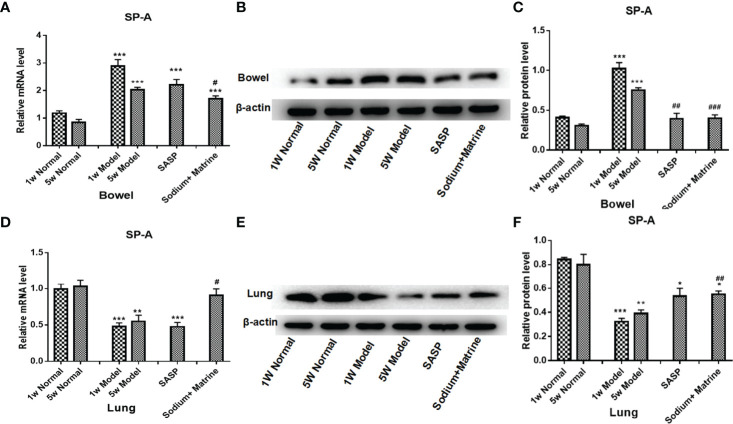
Changes of SP-A in the lung and the bowel. UC models were established with the mucous membrane of colon allergize combine with TNBS-alcohol enteroclysis. **(A)** SP-A mRNA expression in the bowel (PCR); **(B)** SP-A protein expression in the bowel (Western Blotting); **(C)** SP-A relative protein level in the bowel; **(D)** SP-A mRNA expression in the lung (PCR); **(E)** SP-A protein expression in the lung (Western Blotting); **(F)** SP-A relative protein level in lung. Compared with Normal group, *P < 0.05; **P < 0.01; ***P < 0.001. Compared with model group, ^#^P < 0.05, ^##^P < 0.01, ^###^P < 0.001.

The main role of lung surfactant related proteins is to reduce the surface tension of alveoli, maintain the stability of alveoli and normal respiratory function, and the content in the lungs is extremely rich. Among them, surfactant protein A (SP-A) has the largest content, accounting for about 50% of the total amount of SP. SP-A is mainly distributed in the alveolar gas-liquid interface and the bronchial surface that appears to play an important role in mammalian first-line host defense (35), found to have an immune regulatory function. PCR and western-blot results showed that SP-A RNA and the protein in bowel tissues were significantly increased at 1 and 5 weeks after modeling (P<0.001). Sodium Houttuyfonate combined with Matrine treatment could significantly decrease SP-A expression compared with the model group (P<0.05) (**Figures 6A–C**). SP-A RNA and protein in lung tissues were significantly reduced at 1 and 5 weeks after modeling (P<0.001). Sodium Houttuyfonate combined with Matrine treatment could significantly increase SP-A expression compared with the model group (P<0.05) (**Figures 6D–F**). This suggested SP-A was related to the mechanism of UC-related lung injury, and that it was a common material basis between lung and bowel tissues.

### Evaluation of Toxicity of Traditional Chinese Medicine in Rats

**Figure 7:**
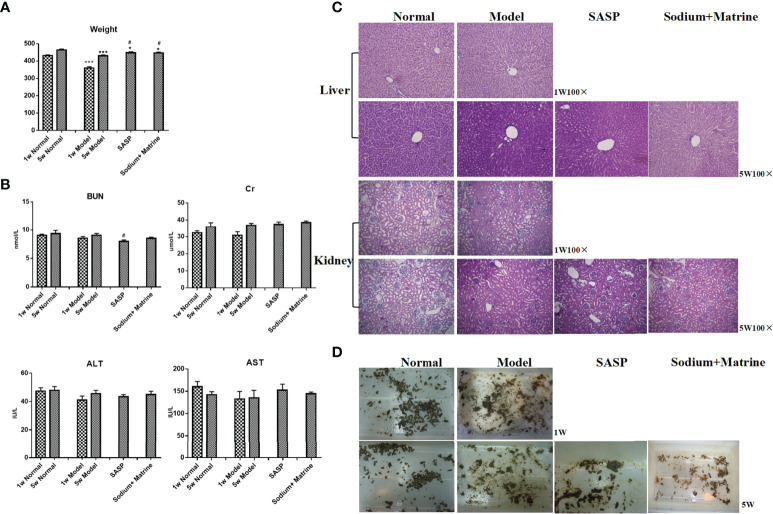
Evaluation of toxicity of Sodium Houttuyfonate combined with Matrine in rats. Ulcerative colitis models were established with mucous membranes of colons sensitized with TNBS-alcohol enteroclysis. Treatments were administered with SASP, Sodium Houttuyfonate combined with Matrine. **(A)** Body weight changes. **(B)** Hepatorenal function changes. ALT, AST, BUN, and Cr were evaluated. **(C)** HE staining of liver and kidney. **(D)** Fecal characteristics. TNBS, immune- 2,4,6-trinitrobenzene sulfonic acid; SASP, sulfasalazine; ALT, alanine aminotransferase; AST, aspartate aminotransferase; BUN, blood urea nitrogen; Cr, Creatinine. and Compared with the model group, ^#^P < 0.05.

A significant decrease in the weight of the model group rats at 1 w and 5 w compared to 1 w and 5 w of the normal group (P < 0.001). The weight of rats in Sodium Houttuyfonate combined with the Matrine group and SASP group was significantly higher than that of rats in the model group (P < 0.05) (**Figure 7A**). The results showed that the weight of rats decreased after modeling and increased after the administration of drugs. There was no significant difference in serum alanine aminotransferase and aspartate aminotransferase in the blood samples of all groups (**Figure 7B**). After 5 w of drug administration, the blood urea nitrogen in the SASP group was significantly lower than that of the model group (P < 0.05); however, no significant difference was observed among the other groups. Negligible deviation occurred in the serum creatinine between each group (P > 0.05) (**Figure 7B**). No significant differences were found in the tissues of the liver and kidney (**Figure 7C**). In addition, we found that the feces of rats in the normal group were black, brown, or granular, without mucus, pus, and blood. The rats in the model group had a purulent and bloody stool, the stool was not formed, the color became lighter, and it was a mucinous stool or loose stool. The feces of each treatment group were mostly shaped, with a small amount of mucus stool or soft stool (**Figure 7D**).

## Discussion

Toll-like receptors (TLRs) are pattern recognition receptors of enter-mucosa inherent immunity recognizing pathogen-associated molecular patterns (PAMPs) (36, 37), mainly monitoring the microbe balance in the enteric cavity, and transferring information to mucosa lamina propria cells (38). The normal intestinal epithelial cells (IECs) express rare TLR2 and TLR4, but TLR4 expression in UC patients is upregulated (39). Ligand binding to TLRs initiates signaling cascades that activate NF-κB, MAPK (40, 41), and interferon response factors (42). The activation of NF-κB induces the expression of inflammatory factors, costimulatory molecules, and ICOS (43), which may interact at the apical surface and induce responses in the intestinal epithelial cell, which in turn produces cytokines, chemokines, and other mediators, inducing inflammatory activation of the mucosal immune system (39). In acute DSS-induced colitis mice, Anselmi et al. demonstrated that mu opioid receptor agonists significantly decreased diarrhea, blood in the stool, and weight loss through suppression of NF-κB expression (44). However, Egan LJ’s study demonstrated the physiological importance of the NF-κB system in protection against radiation-induced death in the intestinal epithelium *in vivo* and identified IKKbeta as a key molecular target for radioprotection in the bowel (45). Indirect blocking of NF-κB activation through lack of the MyD88 gene in intestinal epithelial cells, made the DSS-induced colitis worse in mice (46). In this study, the expression of TLR4 and NF-κB in the bowel of the model group were dropped at 1 w and 5 w, and were slightly increased after treatment with Sodium Houttuyfonate combined with Matrine.

IL-8 is a powerful neutrophil chemotactic factor and active factor, which is mainly produced by mononuclear macrophages, endothelial cells, skin cells, and T cells under the stimulus of IL-1, TNF-α, and lipopolysaccharide (LPS) in the inflammatory reaction, and then causes tissue damage mainly through chemotaxis and activation of neutrophils, and chemotaxis of some basophils and T cells as well. TNF-α is mainly produced by LPS-activated mononuclear macrophages, and in inflammation, promotes neutrophil aggregation and activation, promotes endothelial cell adhesion molecule expression and causes prothrombin effect, and promotes the inflammation to start and continue. In patients with active ulcerative colitis, IL-8 and TNF-α mRNA levels were significantly higher than those in the controls (30). *In situ* hybridization detected increased expression of IL-8 mRNA in macrophages, pericrypt myofibroblasts, and the epithelium of tissue specimens with active lesions of IBD. The secretion of IL-8 from macrophages and myofibroblasts obtained from control patients was upregulated by inflammatory cytokines and bacterial products. TNF-α could promote the secretion of intestinal epithelial cells and IL-8 expression, induce colon epithelial cell apoptosis, and promote UC (47). TNF-α mRNA expression was increased in UC patients, corresponding to the inflammatory activity. TNF-α mRNA expression was increased in UC patients, corresponding to inflammatory activity. Opioids such as Methadone could downregulate TNF-α activities and be beneficial for the maintenance therapy of UC (48). However, DSS-induced inflammation was significantly enhanced in TNF-α -/- mice compared to TNF-α +/+ mice, which suggested that persistent and marked blockage of TNF-α bioactivity might exert a detrimental effect on acute intestinal inflammation (49). In the bowel of the model group, IL-8 increased significantly at 1 w but decreased at 5 w. TNF-α was decreased significantly in the bowel of the model group both at 1 w and 5 w. After the treatment of Sodium Houttuyfonate combined with Matrine, IL-8 increased significantly and TNF-α was slightly upregulated. Sodium Houttuyfonate combined with Matrine could significantly improve intestinal inflammatory response.

MIF is an important proinflammatory factor. It is secreted by a series of immune cells, such as macrophages, dendritic cells, lymphocytes, neutrophils, and pituitary cells (50), and can be induced by LPS interferon γ (IFN-γ) and TNF-α (51). Once MIF is secreted, it regulates a broad range of immune and inflammatory activities, including the induction of inflammatory cytokines, such as IL-1β, IL-6, TNF-α, IFNγ, IL-12, and IL-8 (52). MadCAM-1 is a kind of cell surface adhesion molecule that is selectively expressed in the intestinal mucosa and associated lymphoid tissue of vascular endothelial cells, involved in lymphocyte homing to mucosal sites and cell-cell interactions during the immune response (53). The expression of MAdCAM-1 on gut endothelial was increased in sites of mucosal inflammation in patients with inflammatory bowel disease, but not IBS controls (54). The increased expression of adhesion molecules on endothelial cells was mediated by pro-inflammatory cytokines such as interleukin (IL) 1 and TNF-α (54). Pulmonary surfactant proteins have many roles in surfactant-related functions and innate immunity. One of these proteins is SP-A, which plays a role in both surfactant-related processes and host defense (55), and is the first surfactant protein with an immune-regulatory function that plays an important role in mammalian first-line host defense (56). SP-A is involved in regulating innate pulmonary immune response as a result of interaction with various receptors on the surface of immune cells, including Toll-like receptor-2, Toll-like receptor-4, signal transduction inhibitory regulatory protein A and so on (57). SP-A regulates inflammatory cell response by binding to cell surface pattern recognition receptors. Reduced secretion or functional deficiency of pulmonary surfactant-related proteins is an important factor in lung injury (58).

In this study, we established a rat model of UC by the immune complex method of colonic mucosal sensitization and TNBS—50% ethanol enema. This model had good repeatability and longer duration, which was more in line with the pathogenesis of human UC. In this study, we found that the lung expresses less NF-κB mRNA at 1 week, and more TLR4 mRNA, MIF, and MAdCAM-1 at 5 w. TNF-α and IL-8 were expressed in the lung and increased both at 1 w and 5 w. This suggests that the acute inflammatory of the lung, is mainly caused by TNF-α and IL-8, which is chemotaxis and activation of neutrophils. Sodium Houttuyfonate combined with Matrine could reduce this pulmonary inflammatory response. The decrease in NF-κB during early stage of pneumonia might be caused by cell apoptosis. However, with the decline of cell apoptosis, TLR4 mRNA in the lung increased at 5 w and activated more NF-κB, which had no significant difference compared with the control. MIF mRNA and MAdCAM-1 mRNA also increased remarkably at 5 weeks, which suggests that macrophages participate in lung inflammation and that lymphocytes selectively home to the lung during periods of chronic inflammation. Sodium Houttuyfonate combined with Matrine could effectively reduce the lung homing of lymphocytes and improve lung inflammation. The bowel expressed less NF-κB mRNA and TNF-α both at 1 w and 5 w, and less MIF but more IL-8 at 1 w. MAdCAM-1 expressed in the bowel increased both at 1 w and 5 w. The decrease in NF-κB mRNA and TNF-α might be caused by cell apoptosis, and necrotic tissue was found under the microscope, so they were not damage factors in the colitis. And we found that Sodium Houttuyfonate combined with Matrine could inhibit apoptosis and tissue necrosis, to protect the bowel tract from damage. MIF decreased but IL-8 increased at 1 w, suggesting that macrophages could not work effectively in acute colon inflammation and that too many neutrophils in the colon tissue were damaged. Interestingly, MAdCAM-1 increased both at acute and chronic colitis, suggesting that lymphocytes of the adaptive immune system might be the main damage factors of the bowel in this model. Sodium Houttuyfonate combined with Matrine could effectively inhibit the intestinal expression of MAdCAM-1 and regulate the damage caused by lymphocytes of the adaptive immune system in bowel tissues. The indexes in serum were infected both by the lung and bowel but were not the same as those in the lung or the bowel. TNF-α in the serum decreased at 1 w and might be much influenced by the decrease of TNF-α in the bowel, and the increase at 5 w might be influenced by the increase of TNF-α in the lung. IL-8 in the serum remarkably decreased at 1 w but had no visible difference at 5 w, which suggests that IL-8 chemotaxis neutrophils to the diseased region of both lung and bowel in acute inflammation, but in chronic inflammation just to the lung. After treatment, IL-8 in the serum increased and IL-8 in the lung decreased, indicating that Sodium Houttuyfonate combined with Matrine directly reduced the local inflammatory response of lung tissue, it also transferred part of IL-8 to the serum to indirectly reduce the inflammatory response. MadCAM-1 in the serum increased at 1 w, but had no visible difference at 5 w, it suggested that lymphocytes homing to the periphery immune tissue from the central immune tissue in acute inflammation, but homing to specific organ from periphery immune tissue in chronic inflammation. After treatment, the content of MAdCAM-1 protein in serum increased significantly, and the expression level of MAdCAM-1 mRNA in lung and bowel decreased significantly, which might be Sodium Houttuyfonate combined with Matrine could reduce the homing of lymphocytes in lung and bowel and increase the homing of lymphocytes to peripheral blood and other tissues, to further protect the bowel and lung from injury. SP-A is an important host defense factor in the lung. After the treatment of Sodium Houttuyfonate combined with Matrine, the level of SP-A in the lung increased, and the level of IL-8 in the lung also increased. These results suggest that IL-8 might play a pathogenic role by affecting the expression of SP-A. The specific mechanism needs to be further studied in the later stage.

## Conclusion

In conclusion, the cause of lung injury in this model might be related to inherent immunity and adaptive immunity, presented by the increase of TNF-α, TLR4, IL-8, MIF, and MadCAM-1, and the decrease of SP-A. The cause of bowel injury in this model might be mostly related to adaptive immunity, presented by the increase of MadCAM-1, which homes lymphocytes to diseased regions. Sodium Houttuyfonate combined with Matrine could effectively improve UC and protect UC-related lung injury in immune regulation. This study provides a preliminary theoretical basis for future clinical trials. In addition, we found that the cause of lung injury and bowel injury in this model were incompletely conforming, which suggests that the inflammatory factors in the lung were not transfused from the bowel. There might be an exclusive relationship between the lung and bowel but the specific mechanism needs to be further studied.

## Data Availability Statement

The original contributions presented in the study are included in the article/supplementary material. Further inquiries can be directed to the corresponding author.

## Ethics Statement

The animal study was reviewed and approved by the Animal Experimentation Committee at Beijing University of Chinese Medicine. All animal procedures were performed strictly within national regulations and guidelines.

## Author Contributions

LN and ST conceived and designed the study; LN, SJ, and LZ performed the experiments and collected the data; FW and XY contributed to analyzing the data; LN wrote the manuscript; XW and ST made manuscript revisions. All authors have read and approved the manuscript.

## Funding

This work was supported by grants from the Nation “973” project (2009CB522705), the Natural Science Foundation of China project (81904171), the Jiangsu Postdoctoral Research Foundation (2020Z388), and the Nantong science and technology project (MS12017020-1).

## Conflict of Interest

The authors declare that the research was conducted in the absence of any commercial or financial relationships that could be construed as a potential conflict of interest.

## Publisher’s Note

All claims expressed in this article are solely those of the authors and do not necessarily represent those of their affiliated organizations, or those of the publisher, the editors and the reviewers. Any product that may be evaluated in this article, or claim that may be made by its manufacturer, is not guaranteed or endorsed by the publisher.
